# Cholesterol utilization in mycobacteria is controlled by two TetR-type transcriptional regulators: *kstR* and *kstR2*

**DOI:** 10.1099/mic.0.034538-0

**Published:** 2010-05

**Authors:** Sharon L. Kendall, Philippa Burgess, Ricardo Balhana, Mike Withers, Annemieke ten Bokum, J. Shaun Lott, Chen Gao, Iria Uhia-Castro, Neil G. Stoker

**Affiliations:** 1 Department of Pathology and Infectious Diseases, The Royal Veterinary College, Centre for Emerging, Endemic and Exotic Disease, Hawkshead Lane, Hertfordshire, AL9 7TA, UK; 2 Laboratory of Structural Biology and Maurice Wilkins Centre for Molecular Biodiscovery, School of Biological Sciences, University of Auckland, New Zealand; 3 Department of Environmental Biology, Centro de Investigaciones Biológicas-Consejo Superior de Investigaciones Científicas, 28040 Madrid, Spain

## Abstract

*Mycobacterium tuberculosis* is able to use a variety of carbon sources *in vivo* and current knowledge suggests that cholesterol is used as a carbon source during infection. The catabolized cholesterol is used both as an energy source (ATP generation) and as a source of precursor molecules for the synthesis of complex methyl-branched fatty acids. In previous studies, we described a TetR-type transcriptional repressor, *kstR*, that controls the expression of a number of genes involved in cholesterol catabolism. In this study, we describe a second TetR-type repressor, which we call *kstR2.* We knocked this gene out in *Mycobacterium smegmatis* and used microarrays and quantitative RT-PCR to examine the effects on gene expression. We identified a palindromic regulatory motif for KstR2, showed that this motif is present in three promoter regions in mycobacteria and rhodococcus, and demonstrated binding of purified KstR2 to the motif. Using a combination of motif location analysis, gene expression analysis and the examination of gene conservation, we suggest that *kstR2* controls the expression of a 15 gene regulon. Like *kstR*, *kstR2* and the *kstR2* regulon are highly conserved among the actinomycetes and studies in rhodococcus suggest a role for these genes in cholesterol catabolism. The functional significance of the regulon and implications for the control of cholesterol utilization are discussed.

## INTRODUCTION

The success of *Mycobacterium tuberculosis*, the causative agent of tuberculosis, is partly attributed to its ability to persist within the host (Honer zu Bentrup & Russell, 2001). The antibiotic insensitivity shown by persistent bacilli makes the understanding of the intracellular lifestyle of this pathogen a matter of urgency (Sacchettini *et al.*, 2008). A major advance in understanding the pathogenicity of *M. tuberculosis* was the discovery that the bacterium uses lipids as a carbon source *in vivo.* Evidence for this is based on observations that enzymes involved in fatty acid degradation are induced during infection (Dubnau *et al.*, 2002, 2005; Schnappinger *et al.*, 2003; Tailleux *et al.*, 2008; Talaat *et al.*, 2004) and deletion of genes involved in fatty acid metabolism causes severe attenuation in disease models (McKinney *et al.*, 2000; Munoz-Elias & McKinney, 2005; Munoz-Elias *et al.*, 2006; Sassetti & Rubin, 2003). Despite the body of evidence for the use of lipids as a carbon source during infection, the nature of the lipid(s) utilized has remained elusive and little is known about the genetic regulation of lipid metabolism in mycobacteria.

Recently, we described a transcriptional repressor, *kstR*, that controls the expression of a large regulon involved in lipid metabolism in mycobacteria (Kendall *et al.*, 2007). A study undertaken in the closely related species, *Rhodococcus jostii* RHA1, showed that many of the genes in the *kstR* regulon are induced by the steroid cholesterol and the authors of this study assigned a number of genes in the regulon to the cholesterol degradation pathway (Van der Geize *et al.*, 2007). Subsequent biochemical and structural studies have confirmed a role for these genes specifically in cholesterol catabolism (Capyk *et al.*, 2009; Knol *et al.*, 2008; Lack *et al.*, 2009; Yam *et al.*, 2009). The observation that many of the genes in the *kstR* regulon are also induced *in vivo* or are essential for virulence highlights the importance of cholesterol catabolism in the pathogenicity of *M. tuberculosis* (Brzostek *et al.*, 2007; Chang *et al.*, 2007; Hu *et al.*, 2010; Nesbitt *et al.*, 2010; Rengarajan *et al.*, 2005; Sassetti & Rubin, 2003; Schnappinger *et al.*, 2003; Yam *et al.*, 2009).

The study by Van der Geize *et al*. (2007) identified six clusters of genes that were upregulated in response to cholesterol in *R. jostii.* One of these clusters corresponds to the *kstR* regulon. In this study, we describe a second transcriptional repressor, *Rv3557c* (*kstR2*), that controls the expression of a subset of genes within the *kstR* cluster. This work further informs how the genes involved in cholesterol catabolism are controlled in mycobacteria and related actinomycetes. The observation that a number of genes in the *kstR2* regulon are induced in macrophages or are essential for infection (Rengarajan *et al.*, 2005; Sassetti & Rubin, 2003; Schnappinger *et al.*, 2003) means that this work also informs the regulatory networks utilized by mycobacteria and other pathogenic actinomycetes for survival *in vivo.*

## METHODS

### Bacterial strains and culture conditions.

The strains and plasmids used in this study are described in Table 1. All bacterial cultures were grown at 37 °C and liquid cultures were grown with shaking (200 r.p.m.). *Escherichia coli* DH5*α* was used as a strain for cloning and *E. coli* BL21 (DE3) was used as a host for expression. Both *E. coli* strains were grown in Luria–Bertani medium. *Mycobacterium smegmatis* mc^2^155 was grown in Middlebrook 7H9 broth (Difco) containing 10 % oleic acid–albumin–dextrose–catalase supplement (OADC; Becton Dickinson) and 0.05 % Tween 80 or Middlebrook 7H11 agar containing 10 % OADC. Hygromycin (50 μg ml^−1^), kanamycin (50 μg ml^−1^ for *E. coli* and 20 μg ml^−1^ for *M. smegmatis*), 5-bromo-4-chloro-3-indolyl-*β*-d-galactopyranoside (Xgal, 50 μg ml^−1^) and sucrose (2 % w/v) were used for selection as appropriate.

### Deletion of *kstR2*_Msm_.

A 505 bp deletion was made in *kstR2*_Msm_ by homologous recombination (Parish *et al.*, 1999). Briefly, a 3.5 kb fragment was amplified from genomic DNA using Δ*kstR2*_Msm_ forward and reverse primers (Table 2) and cloned into pUC18 using the *Kpn*I sites designed within the primers. The resulting plasmid, pPB1, was used as a template in an inverse PCR with inv_*kstR2*_Msm_ forward and reverse primers. *Eco*RV digestion and religation of the resulting PCR fragment created plasmid pPB2 containing the 505 bp deletion in *kstR2*_Msm_. Finally, a 3 kb fragment containing Δ*kstR2*_Msm_ was subcloned from pPB2 into p2NIL (pPB3) and the *Pac*I cassette from pGOAL19 was inserted into pPB3 resulting in the suicide delivery vector pPB4.

pPB4 was electroporated into *M. smegmatis* mc^2^155 and single crossovers were selected for using kanamycin, hygromycin and Xgal. A single antibiotic-resistant blue colony was taken and restreaked onto 7H11 with no selection and allowed to grow for 3–5 days. Double crossovers were selected for by plating serial dilutions onto 7H11 plates containing Xgal and sucrose. Potential double crossovers (white sucrose-resistant colonies) were screened using colony PCR. The resulting mutant was called ΔkstR2_Msm_. The procedure was repeated in a ΔkstR_Msm_ background to make a Δ*kstR/*Δ*kstR2* double mutant. The whole mutagenesis and selection procedure is not affected by the ΔkstR_Msm_ background as this is an unmarked mutant.

### RNA extraction.

RNA extractions were done by direct sampling into guanidinium thiocyanate (GTC). A 10 ml volume of an exponential culture (OD_600_ 0.4–0.5) was added to 40 ml 5 M GTC. The culture was pelleted by centrifugation (20 min, 4000 ***g***, 4 °C) and resuspended in 200 μl water. A 700 μl volume of buffer RLT (Qiagen) was added to the resuspended pellet and the bacteria were lysed in screw-capped tubes containing 0.5 ml 0.1 mm zirconia/silica beads (Biospec) using a Precellys system (Stretton Scientific). Cell lysates were recovered by centrifugation (5 min, 13 000 ***g***, 4 °C) and RNA purified from the lysate using an RNeasy kit (Qiagen) according to the manufacturer's instructions. A DNase treatment was done ‘on the column’ according to the manufacturer's instructions and the samples were eluted in 30 μl RNase-free water. Finally, the quality and quantity of the RNA was assessed using a NanoDrop (NanoDrop technologies).

### Reverse transcription reactions for quantitative RT-PCR (RTq-PCR).

RNA was given a second round of DNase treatment (Invitrogen) for 30 min at 37 °C prior to reverse transcription. RNA (100 ng) was reverse transcribed in a total volume of 20 μl with 300 ng random primers (Invitrogen), 10 mM DTT, 0.5 mM each of dCTP, dATP, dGTP and dTTP, and 200 units Superscript III reverse transcriptase (Invitrogen). Primers were annealed by heating to 65 °C for 10 min and then snap-cooled on ice before adding the remaining components. Reverse transcription took place at 55 °C for 50 min.

### RTq-PCR.

RTq-PCRs were done using the DyNAmo SYBR Green qPCR kit (MJ Research) and performed using the DNA Engine Opticon 2 System (GRI). A 1 μl volume (equivalent to 5 ng RNA) of cDNA was used in the reactions which also contained 1× DNA master mix, 0.3 μM of each primer in a total reaction volume of 20 μl. The sequences of the primers used are given in Table 2. Reactions were heated to 95 °C for 10 min before cycling for 35 cycles of 95 °C for 30 s, 62 °C for 20 s, and 72 °C for 20 s. An 80 °C melt was done at the end of each cycle to ensure that primer dimers had melted before fluorescence due to PCR product was captured. The specificity of the PCR product was ensured by doing melting curve analysis and running the fragment on a gel at the end of each PCR. The experiment was performed in triplicate and each gene was measured in duplicate, giving a total of six data points per gene.

### Expression and purification of recombinant KstR2_Mtb._.

The *kstR2*_Mtb_ gene was cloned into the pET30a expression vector using *Nco*I and *Hin*dIII sites. The resulting plasmid, pSK49, was sequence verified and used for expression and purification of C-terminally His-tagged KstR2_Mtb_. For expression, *E. coli* BL21(DE3) cultures containing plasmid pSK49 were grown at 37 °C until mid-exponential phase. Cultures were induced with 1 mM IPTG for 2 h at 37 °C and harvested by centrifugation (10 min, 4000 ***g***, 4 °C). The cell pellet was resuspended in 5 ml lysis buffer (20 mM HEPES pH 8.0, 150 mM NaCl, 1 mM *β*-mercaptoethanol, 10 mM imidazole) and lysed by passage through a cell disrupter (Constant Systems) set at 18 000  p.s.i. (124.2 MPa). The lysate was centrifuged (25 min, 16 000 ***g***, 4 °C) and His6-KstR2_Mtb_ from the soluble fraction was purified by immobilized metal ion affinity chromatography using a HiTrap Ni-NTA column (GE Healthcare Biosciences), followed by size exclusion chromatography using a Superdex 200 10/30 column (GE Healthcare Biosciences).

### Electrophoretic mobility shift assays (EMSAs).

Untagged recombinant KstR2_Mtb_ was used in EMSAs. The tag was removed by cleavage with recombinant tobacco etch virus protease. Single-stranded oligonucleotide probes (30-mer) were annealed in shift buffer, consisting of 20 mM Tris/HCl (pH 8.0), 10 mM MgCl_2_ and 75 mM NaCl by heating to 95 °C for 10 min and cooling slowly to room temperature. The resulting double-stranded oligonucleotide probes were incubated at a final concentration of 1 μM for 20 min at 37 °C with recombinant KstR2_Mtb_ at a final concentration of 6 μM. Reaction mixtures were loaded onto an 8 % polyacrylamide gel in 0.5× TBE buffer (45 mM Tris-borate, 1 mM EDTA). After electrophoresis, gels were stained for 1 h at room temperature in 0.5× TBE buffer with 0.04 mg ethidium bromide ml^−1^.

For the competition experiments, the double-stranded oligonucleotide probes were end-labelled with DIG-11-ddUTP using the DIG gel shift kit, second generation (Roche), according to the manufacturer's instructions. For the binding reaction, 0.03 pmol labelled probe in shift buffer, with the addition of 0.1 mg poly-l-lysine and 1 mg poly[d(I-C)] ml^−1^, was incubated with 3 pmol of recombinant KstR2_Mtb_. Specific and non-specific competitors were added for the control reactions. Specific competition reaction mixtures contained a 150-fold excess of unlabelled probe, and non-specific competion mixtures contained an excess of poly[d(I-C)]. Incubations were carried out for 20 min at 37 °C, and reaction mixtures were loaded onto 8 % polyacrylamide gels in 0.5× TBE buffer. After electrophoresis, the DNA–protein complexes were contact blotted onto positively charged Hybond-N+ nylon membranes (Amersham), and detected by anti-DIG-alkaline phosphatase and the chemiluminescent substrate CSPD, as described by the manufacturer (Roche). Membranes were exposed to X-ray film at room temperature for 10–30 min.

### Microarray analysis of *M. smegmatis* Δ*kstR2*.

Microarrays were provided by the Pathogen Functional Genomics Resource Centre at TIGR (http://pfgrc.jcvi.org/). Wild-type RNA was hybridized against mutant RNA and the experiments were performed with biological triplicates and technical replicates that included a dye-swap. The labelling and hybridizations were performed as described previously (Kendall *et al.*, 2007). Briefly, 2–10 μg RNA was labelled with either Cy3-dCTP or Cy5-dCTP (Amersham Pharmacia Biotech) in a reaction containing 3 μg random primers, 0.5 mM each of dATP, dGTP and dTTP, 0.2 mM dCTP, 10 mM DTT, 60 μmol Cy3-dCTP (or Cy5-dCTP) and 500 units Superscript II (Invitrogen Life Technologies). Samples were purified using a MinElute PCR Purification kit from Qiagen and hybridized onto the array in hybridization buffer (4× SSC, 40 % formamide, 0.1 % SDS). Hybridization took place at 65 °C for 16 h and slides were washed in a series of buffers (Buffer 1: 1× SSC, 0.05 % SDS, 65 °C; Buffer 2: 0.06× SSC, room temperature). Finally slides were dried by centrifugation (1500 ***g***, 5 min, room temperature) and scanned (Affymetrix 418 scanner).

### Microarray data analysis.

Data analysis was performed using functions from the  μG@Sbase microarray data analysis pipeline (http://www.bugs.sghms.ac.uk/bugsbase/), limma (linear models for microarray data analysis) (Smyth *et al.*, 2005; http://bioinf.wehi.edu.au/limma/) and yasma (Wernisch *et al.*, 2003) software packages. Data for duplicate spots within the arrays were averaged before the linear model fit was performed. False discovery rate adjustment was made (Benjamini, 1995) and genes were considered significant if they had an adjusted *P*-value less than 0.05.

### Bioinformatic analyses.

Comparative genomics and genome browsing were performed using ACT (Carver *et al.*, 2005). Sequence similarity searches were performed using blast (Ladunga, 2002). Sequence alignments were performed using clustal w (Thompson *et al.*, 1994) and motif analysis was carried out using MEME and MAST (Bailey & Elkan, 1994; Bailey & Gribskov, 1998).

## RESULTS

### Comparative genomics of the *kstR2* region in *M. smegmatis, M. tuberculosis* and *R. jostii*

**Fig. 1.**
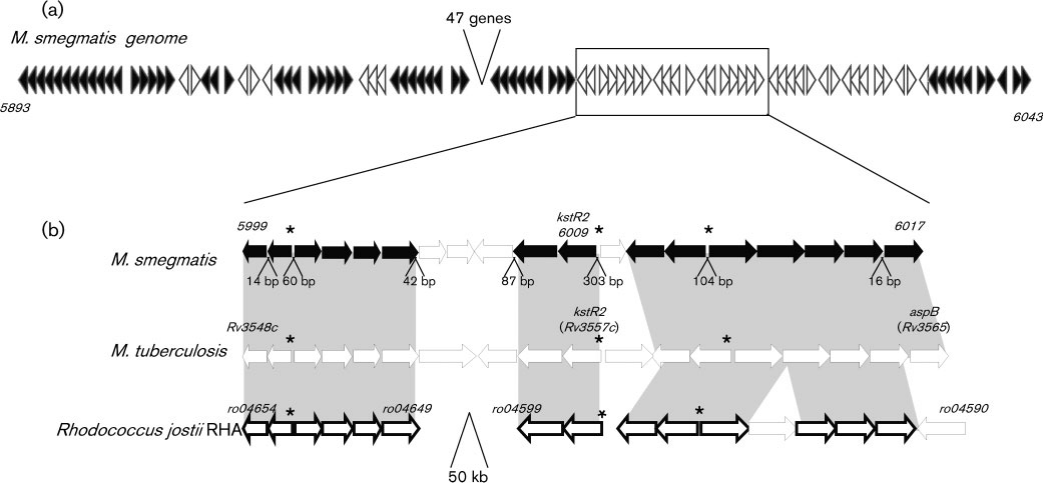
The putative *kstR2* regulon in *M. smegmatis*. (a) Representation of the *M. smegmatis* genome from *MSMEG_5893* to *MSMEG_6043*. The directions of the arrows represent gene direction and the arrows filled in black are genes controlled by *kstR* (Kendall *et al.*, 2007). (b) The magnified region corresponds to *MSMEG_5999* to *MSMEG_6017* and the bottom two lines of arrows correspond to the *M. tuberculosis* and *R. jostii* RHA1 genomes. Orthologous genes are indicated by the grey shaded areas. Although *MSMEG_6010* and *Rv3558* have a conserved context (neighbouring genes are conserved) there is no homology between these two genes and they are not considered to be orthologues. The locations of the KstR2 motif are indicated by asterisks and the sizes of the intergenic regions in the *M. smegmatis* genome are shown. Genes indicated by thickly outlined arrows in the rhodococcal genome have been shown previously to be induced by cholesterol (Van der Geize *et al.*, 2007). Genes indicated by arrows filled in black in the *M. smegmatis* genome are those in the *kstR2* regulon.

In *M. smegmatis*, *kstR* controls the expression of 83 genes (Kendall *et al.*, 2007). Fifty-five of these *kstR*-controlled genes lie within a particular region in the *M. smegmatis* genome (*MSMEG_5893–MSMEG_6043*) (Fig. 1a). This region is highly conserved in *M. tuberculosis* (*Rv3492c*–*Rv3574*) and *R. jostii* (*ro4482*–*ro04705*), and studies in *R. jostii* have shown that all of the *kstR*-controlled genes within the *ro4482–ro04705* region are induced by cholesterol (Van der Geize *et al.*, 2007). However, not all of the cholesterol-induced genes in this region are controlled by *kstR*. Analysis of the *R. jostii* genome shows that there are three possible operons (*ro04654–ro04649*, *ro04599–ro04598* and *ro04597–ro04591*) that are induced by cholesterol but not controlled by *kstR* (Fig. 1b, bottom line). These genes are conserved in *M. tuberculosis* (*Rv3548c–Rv3565*, Fig. 1b, middle line) and *M. smegmatis* (*MSMEG_5999–MSMEG_6017*, Fig. 1b, top line), and examination of the function of the genes in this region shows that there is another TetR-type regulator which we are calling *kstR2* (*ro04598*, *Rv3557c*, *MSMEG_6009*).

Given that these genes are induced by cholesterol in *R. jostii*, but are not part of the *kstR* regulon, and that genes are often controlled by regulators in the near vicinity, we hypothesized that *kstR2* controls the expression of genes within this cluster. In order to test this, we searched for possible regulatory motifs firstly within its own promoter region, and then on a genome-wide scale.

### Identification of a potential KstR2 regulatory binding motif

**Fig. 2.**
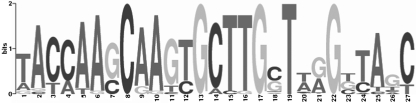
Sequence logo of the KstR2 binding motif. Sequence logos (Crooks *et al.*, 2004) show the relative frequency of each base at each position of the motif. The *y*-axis shows the information content.

Examination of the genomes of other closely related actinomycetes (*Mycobacterium avium* subsp. *paratuberculosis*, *Mycobacterium avium*, *Mycobacterium marinum*, *Nocardia farcinica* and *Rhodococcus equi*) using ACT and clustal w showed that *kstR2* is conserved in these species with all orthologues showing over 50 % amino acid identity to the *M. tuberculosis* protein (data not shown). In order to identify a potential regulatory motif for KstR2, we used the promoter regions of the *kstR2* orthologues as a training set for the motif identification program MEME (Bailey & Elkan, 1994). This identified a potential regulatory sequence that contains a 14 bp inverted palindromic motif **A**n**CAAG**nn**CTTG**n**T** (Fig. 2). Palindromic DNA binding regions are a common feature of TetR-type regulators and this motif is similar in structure to the KstR motif, which is also a 14 bp inverted palindrome. In order to determine if the motif is present elsewhere in the genomes of *M. tuberculosis*, *M. smegmatis* and *R. jostii*, we searched a database of intergenic regions using MAST (Bailey & Gribskov, 1998). This predicted three motifs in each genome that were all situated within the intergenic regions of divergently transcribed genes near to *kstR2*, and are shown in Fig. 1 and Table 3.

### KstR2_Mtb_ binds to the conserved regulatory motif

**Fig. 3.**
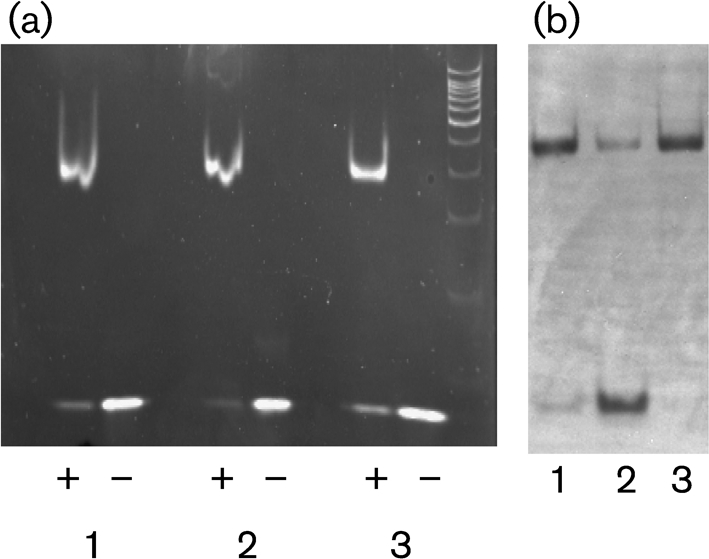
Binding of purified KstR2_Mtb_ to 30 bp probes containing the motif. (a) EMSA of purified KstR2_Mtb_ to 30 bp probes from the three intergenic regions (*Rv3549c–Rv3550*, *Rv3557c–Rv3558* and *Rv3560c–Rv3561*). Lanes: 1, *Rv3549c–Rv3550*; 2, *Rv3557c–Rv3558*; 3, *Rv3560c–Rv3561*. +, with protein; −, without protein. (b) Specific binding of purified KstR2_Mtb_ to a 30 bp probe from the *Rv3557c–Rv3558* intergenic region. Lanes: 1, labelled probe with protein; 2, labelled probe with protein and with 150-fold excess unlabelled probe; 3, labelled probe with protein and with an excess of poly[d(I-C)].

In order to determine whether KstR2 binds directly to the motif that was identified, the protein from *M. tuberculosis* (KstR2_Mtb_) was expressed, purified and used in EMSAs. Probes (30 bp) from the three intergenic regions (*Rv3549c–Rv3550*, *Rv3557c–Rv3558* and *Rv3560c–Rv3561*) containing the motif sequences were used in the assay (Table 2, the locations of the motifs are underlined); the results are shown in Fig. 3. The presence of the purified protein clearly retarded the movement of all the 30 bp probes through the gel, indicating binding of the KstR2_Mtb_ protein to the motifs in each of the regions (Fig. 3a). In order to determine whether the binding was specific, competition assays were used with non-specific and specific competitors (Fig. 3b). The binding was prevented by adding a 150-fold excess of unlabelled probe (Fig. 3b, lane 2) but was not altered by the addition of the non-specific inhibitor poly[d(I-C)] (Fig. 3b, lane 3). The results of these experiments clearly show that purified KstR2_Mtb_ binds specifically to the motif within the three intergenic regions.

### Expression analysis of Δ*kstR2*_Msm_ and defining the *kstR2* regulon

In order to identify the genes controlled by *kstR2*, a 505 bp deletion of *kstR2* was made in the *M. smegmatis* genome using homologous recombination (Parish *et al.*, 1999). The mutagenesis was verified by PCR and sequencing as described in Methods (data not shown). Microarrays were used to determine the effects of knocking out *kstR2*_Msm_ on gene expression. RNA from wild-type and mutant bacteria was labelled with Cy dyes and competitively hybridized onto oligonucleotide arrays. The results are shown in Table 4. Using a *P*-value cut-off of 0.05, a total of eight genes were significantly upregulated and three genes were downregulated (Table 4). All of the significantly upregulated genes were from the region *MSMEG_5999* to *MSMEG_6017* and were associated with the motif identified (Fig. 1).

In order to propose a regulon for *kstR2*, we have combined the microarray data with bioinformatic analysis and have taken motif location, genomic arrangement (the likely operon structure deduced from gene direction and the distances between genes), gene conservation and de-repression by cholesterol in *R. jostii* into account. The *kstR2* regulon is defined below.

### Defining the *kstR2* regulon

The microarray analysis shows that *MSMEG_5999* to *MSMEG_6004* are highly de-repressed in the mutant (Table 4). There are no intergenic gaps in the run of genes *MSMEG_6001* to *MSMEG_6004* and only a 14 bp gap upstream of *MSMEG_5999* (Fig. 1), suggesting that *MSMEG_6001* to *MSMEG_6004* and *MSMEG_6000* to *MSMEG_5999* are transcribed in two divergent operons. A motif is present in the 60 bp intergenic region between *MSMEG_6000* and *MSMEG_6001*, and the orthologous genes are de-repressed in *R. jostii* by cholesterol (Fig. 1). This, together with the demonstration that KstR2_Mtb_ binds to the motif located between the orthologous *M. tuberculosis* genes (Fig. 3a), strongly suggests that KstR2 divergently represses these genes and that this repression is alleviated by cholesterol.

The results suggest that *MSMEG_6005*, *MSMEG_6006* and *MSMEG_6007* are not regulated by KstR2. Firstly, the microarray analysis shows that these genes are not highly or significantly de-repressed in the mutant (Table 4). Secondly, these genes are not conserved in *R. jostii* (or *M. tuberculosis*) so they are unlikely to be required for cholesterol degradation (Fig. 1). There is a 42 bp intergenic region upstream of *MSMEG_6005* and an 87 bp intergenic region upstream of *MSMEG_6007*, which would allow for the presence of an independent promoter, but there is no associated *kstR2* motif (Fig. 1). Taken together, the data suggest that these genes are not under the control of KstR2.

The microarray data show *kstR2* (*MSMEG_6009*) as being downregulated in the mutant strain because this gene is deleted in the mutant. The gene downstream of *kstR2*, *MSMEG_6008*, however, is highly and significantly de-repressed in the mutant strain (Table 4) and there is no intergenic gap between *kstR2* and *MSMEG_6008*, suggesting they are co-transcribed. There is a motif in the 303 bp gap upstream of *kstR2* to which KstR2 binds (Fig. 3a) and the orthologues of both genes are de-repressed by cholesterol in *R. jostii* (Fig. 1). This suggests that *kstR2* and *MSMEG_6009* are expressed as an operon under the control of KstR2.

The run of genes *MSMEG_6011* to *MSMEG_6017* appear to be arranged into two divergent operons: *MSMEG_6012* to *MSMEG_6011* and *MSMEG_6013* to *MSMEG_6017* (Fig. 1). There is a motif in the 104 bp intergenic region between *MSMEG_6012* and *MSMEG_6013* to which KstR2 binds (Fig. 3a). Microarray analysis suggests that these genes are de-repressed in the mutant (Table 4). Levels were not high or significant for *MSMEG_6013* and *MSMEG_6014* in this microarray experiment, but de-repression was confirmed by RTq-PCR (data not shown). All these genes (with the exception of *MSMEG_6017* for which there is no rhodococcal orthologue) are de-repressed by cholesterol in *R. jostii*. Taken together, the data suggest that KstR2 binds to the motif in the intergenic region between *MSMEG_6012* and *MSMEG_6013* to divergently repress expression and that repression is alleviated by cholesterol.

Finally, the microarray analysis suggests that *MSMEG_6010* is not de-repressed in the mutant strain (Table 4) and we have used RTq-PCR to confirm this result (data not shown). There is no orthologue in *M. tuberculosis* or *R. jostii* so this gene is unlikely to be required for cholesterol degradation (Fig. 1). This suggests that KstR2 binds to the motif within the intergenic region between *kstR2* and *MSMEG_6010* to cause repression in one direction only. This is in contrast with the other promoter regions where both divergently located genes are repressed by binding of KstR2 to the motif. Consistent with this, the distance between the putative start of *MSMEG_6010* and the *kstR2* motif is large (285 bp) in comparison with the rest of the motif to start site distances (*MSMEG_6000*, 22 bp; *MSMEG_6001*, 39 bp; *MSMEG_6009*, 22 bp; *MSMEG_6012*, 23 bp; and *MSMEG_6013*, 82 bp). Additionally, a recent study has shown that in *M. tuberculosis kstR2* and *Rv3556c* are de-repressed by cholesterol but *Rv3558c* (*PPE64*) is not (Nesbitt *et al.*, 2010).

In addition to the genes within the *MSMEG_5999* to *MSMEG_6017* region, three genes were significantly downregulated (Table 4). However, none of these genes was associated with a motif and downregulation is likely to be due to secondary effects of the *kstR2* deletion rather than direct control. In summary, the data show that KstR2 controls the expression of 15 genes within the *MSMEG_5999* to *MSMEG_6017* region. These genes are in black in Fig. 1 and in bold type in Table 4.

### KstR2 and KstR act independently of each other

**Fig. 4.**
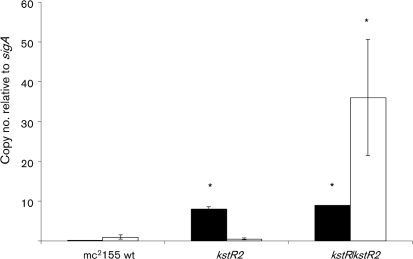
Expression levels of *MSMEG_6001* and *MSMEG_6038* in a wild-type, Δ*kstR2_Msm_* mutant and Δ*kstR/*Δ*kstR2_Msm_* mutant background. The expression levels were measured in mid-exponential-phase aerated cultures using RTq-PCR as described in Methods. The results are expressed relative to *sigA* which was not significantly different (unpaired Student's *t*-test; *P*>0.05) in the mutants compared with the wild-type. Levels of *sigA* expression for the wild-type, Δ*kstR2_Msm_* and Δ*kstR/*Δ*kstR2_Msm_* were 90 495 (±10 458), 89 635 (±4929) and 75 180 (±3818) copies (μl cDNA)^−1^, respectively. Significantly upregulated (de-repressed) genes in the mutants compared with the wild-type are marked with asterisks (unpaired Student's *t*-test; *P*<0.05). Error bars represent ±1 sd. Black bars, *MSMEG_6001* (a KstR2-regulated gene); white bars, *MSMEG_6038* (a KstR-regulated gene).

In order to test whether there is any interaction between the two regulators, we decided to measure the expression of KstR- and KstR2-controlled genes in a wild-type, Δ*kstR2* and Δ*kstR/*Δ*kstR2* double mutant background of *M. smegmatis* using RTq-PCR. The Δ*kstR/*Δ*kstR2* mutant was made by deleting *kstR2* in a ΔkstR_Msm_ mutant that has been described previously (Kendall *et al.*, 2007). We chose to focus on *MSMEG_6001* (KstR2-regulated) and *MSMEG_6038* (KstR-regulated), because they were highly de-repressed in their respective mutant backgrounds (see Table 4 and Kendall *et al.*, 2007) and would therefore act as good reporters. The results, which are given in Fig. 4, show that (as expected) both genes are repressed in the wild-type strain mc^2^155. The *kstR2*-regulated gene *MSMEG_6001* is de-repressed in the Δ*kstR2*_Msm_ mutant but the level of de-repression does not increase when *kstR* is also absent. Additionally, knocking out *kstR2* has no effect on the expression levels of *MSMEG_6038*, a *kstR*-regulated gene. These results clearly show that there is no cross-talk between the regulators.

## DISCUSSION

Previous studies have identified a TetR repressor, *kstR*, that controls a number of genes that are de-repressed by growth on cholesterol (Kendall *et al.*, 2007; Van der Geize *et al.*, 2007). In this study, we show that a second TetR-type repressor, *kstR2*, controls the expression of a small regulon that may also play a role in the utilization of cholesterol in mycobacteria. This second regulon, which consists of 15 genes, is located in the same region as the *kstR* regulon and is de-repressed in *R. jostii* by growth in cholesterol (Fig. 1 and Table 4). The expression of the genes in the *kstR2* regulon is not affected by a deletion in *kstR*; therefore the two regulators act independently of each other. Both KstR and KstR2 negatively autoregulate themselves. This is a common feature of gene regulation and occurs in over 40 % of the transcription factors in *E. coli* (Shen-Orr *et al.*, 2002). Negative autoregulation is known to speed up response time, reduce stochastic gene expression noise and be metabolically economical for the cell (Nevozhay *et al.*, 2009).

The precise roles of each of the genes in the *kstR* or *kstR2* regulons in cholesterol catabolism have yet to be fully defined. Although it is clear that both regulons are de-repressed by cholesterol, not all of the genes may be involved in the degradative pathway. KstR and KstR2 co-ordinately control over 70 genes that are all de-repressed by growth on cholesterol. Van der Geize *et al.* (2007) assigned 28 mycobacterial genes (27 of which belong to the *kstR* regulon) to cholesterol degradation based on sequence identity with other steroid-degrading species. However, only three of the *M. tuberculosis* enzymes have been demonstrated biochemically to catalyse steps in the degradation pathway. This means that the precise functions of many of the genes in both regulons remain undefined. It is possible that the genes may be involved in the assimilation of the products of cholesterol degradation. Cholesterol catabolism generates propionyl-CoA which has been shown to be incorporated into complex methyl-branched chain fatty acids found in the mycobacterial cell wall (Yang *et al.*, 2009). Therefore, it is possible that some of the genes in the *kstR* and *kstR2* regulons are involved in these synthetic pathways. The observation that arylamine *N*-acetyltransferase in *M. tuberculosis* (TBNAT), one of the genes in the *kstR* regulon in *M. tuberculosis* (although not in *M. smegmatis*), is able to utilize propionyl-CoA provides evidence for this (Lack *et al.*, 2009).

Recent *in vivo* studies in *M. tuberculosis* provide contradictory information about the temporal requirement for cholesterol catabolism. Δ*hsaC*, Δ*igr* (*Rv3540c*–*Rv3545c*), Δ*kshA* and Δ*kshB* mutants (which are all *kstR*-regulated genes) are attenuated in the early stages of infection (Chang *et al.*, 2009; Hu *et al.*, 2010; Yam *et al.*, 2009). However, deletion of the cholesterol importer (*mce4*) and another *kstR*-regulated gene, *fadA5*, results in attenuation in the persistent stage of infection (Nesbitt *et al.*, 2010; Pandey & Sassetti, 2008). Studies *in vitro* have shown that the propionyl-CoA generated from cholesterol catabolism only becomes a significant source of propionate in the absence of sugar carbon sources (Yang *et al.*, 2009). Therefore, it is possible that, although the potential to metabolize cholesterol is present throughout infection, the contribution to bacterial metabolism only becomes significant in the later stages of infection when sugar sources of carbon may have run out. The fact that the cholesterol catabolism genes are controlled by two transcriptional regulators may have implications for the temporal expression of each of the regulons during infection.

The degradation of cholesterol and the subsequent elevation of propionyl-CoA levels is a double-edged sword. While propionyl-CoA is a useful metabolite, particularly for the biosynthesis of cell wall fatty acids, accumulation of propionate is also toxic. The accumulation of toxic metabolites has been suggested to be the cause of the early attenuation seen in some of the mutants mentioned above (Chang *et al.*, 2009; Yam *et al.*, 2009). However, it is thought not to account for the early attenuation seen in the Δ*kshA* and Δ*kshB* mutants, as the addition of cholesterol to these mutants growing in glucose does not result in inhibition (Hu *et al.*, 2010). The addition of cholesterol to wild-type cultures grown in glucose also does not result in growth inhibition. In this case, we would expect de-repression of the genes in the *kstR* and *kstR2* regulons enabling the wild-type bacteria to fully metabolize cholesterol. Similarly, the Δ*kstR* and Δ*kstR2* mutants are able to fully metabolize cholesterol as the genes in the regulons are constitutively de-repressed.

Sequencing of the *M. tuberculosis* genome revealed approximately 250 genes involved in lipid metabolism but the precise metabolic pathways in which these genes function have yet to be dissected (Camus *et al.*, 2002; Cole, 1999). Identifying regulons (i.e. subsets of genes that are co-regulated) helps to assign genes to specific metabolic pathways. The work presented here on *kstR2* and our previous studies with *kstR* have identified two regulons, and other laboratories have shown that these genes are de-repressed by growth on cholesterol. The observation that there are (at least) two independently acting regulators involved in cholesterol catabolism invites a number of interesting questions regarding the role of each regulator in pathogenesis. Are the regulons switched on in response to cholesterol simultaneously? Do the regulators recognize and bind to the same ligand? Is there biological significance in this dual regulation or is it just a remnant of how this pathway evolved? Further experiments are required to answer these questions.

## Figures and Tables

**Table 1.** Bacterial strains and plasmids used in this study

**Strain/plasmid**	**Genotype/description**	**Source/reference**
**Strains**		
*E. coli*		
DH5*α*	*supE44* Δ*lacU169* (*φlacZ*ΔM15) *hsdR17 recA1 endA1 gyrA96 thi-*1 *relA1*	Invitrogen
BL21(DE3)	*ompT hsdS*_B_( 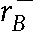 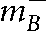 ) *gal dcm* (DE3)	Novagen
*M. smegmati*s		
mc^2^155	High-frequency transformation mutant ATCC 607	Snapper *et al.* (1990)
Δ*kstR2_Msm_*	Δ*kstR2*_Msm_	This study
**Plasmids**		
p2NIL	Gene manipulation vector, *Kan*	Parish & Stoker (2000)
pGOAL19	*Pac*I cassette vector, *hyg* P_ag85_–*lacZ* P_hsp60_–*sacB Amp*	Parish & Stoker (2000)
pET30a	*E. coli* expression vector, *Kan*	Novagen
pUC18	*E. coli* cloning vector, *Amp*	Sambrook & Russell (2001)
pPB1	3.5 kb fragment containing *kstR2*_Msm_ in pUC18, *Amp*	This study
pPB2	505 bp deletion of *kstR2*_Msm_ in pPB2, *Amp*	This study
pPB3	3.0 kb fragment containing Δ*kstR2*_Msm_ in p2NIL, *Kan*	This study
pPB4	pPB3 with the pGOAL19 cassette inserted, *Kan*, *Hyg*	This study
pSK49	*kstR2*_Mtb_ in pET30a expression vector, *Kan*	This study

**Table 2.** Primers used in this study

**Primer**	**Orientation**	Sequence*	**Purpose**
Δ*kstR2*_Msm_	Forward	CCGGTACCGGCATCGGCTCGACCA	Cloning *kstR*_Msm_ into pUC18
	Reverse	CCGGTACCGTCAGAAAGCTTTCATCACGCG	Cloning *kstR*_Msm_ into pUC18
inv_*kstR2*_Msm_	Forward	GGGATATCCAGCAGTACCTCGCCATCGTT	Making *kstR*_Msm_ deletion
	Reverse	GGGATATCAGCAGCTCATCGCGTCTGCTAGC	Making *kstR*_Msm_ deletion
pET_*kstR2*_Mtb_	Forward	AGTCCATGGGG**ATG**GATCGAGTG	Cloning *kstR2*_Mtb_ into pET30a for expression
	Reverse	CGGAAGCTT**TCA**GACTCCCTCTTT	Cloning *kstR2*_Mtb_ into pET30a for expression
*MSMEG_6001*	Forward	CTCGGTCACCGTGAACTACC	RTq-PCR expression analysis
	Reverse	TCTGCATCTCCTTGATCTGTCG	RTq-PCR expression analysis
*MSMEG_6038*	Forward	TCGATGAGATCGGCTTCTTC	RTq-PCR expression analysis
	Reverse	CAGTTGTGCACACCGATGAT	RTq-PCR expression analysis
*MSMEG_2758* (*sigA*)	Forward	CCAAGGGCTACAAGTTCTCG	RTq-PCR expression analysis
	Reverse	CTTGTTGATCACCTCGACCA	RTq-PCR expression analysis
*Rv3549c/Rv3550*	Forward	GCGTACCAAGCAAGTGCTTGCTTAGGTAGC	Oligonucleotides used in EMSAs
	Reverse	GCTACCTAAGCAAGCACTTGCTTGGTACGC	Oligonucleotides used in EMSAs
*Rv3557c/Rv3558*	Forward	GCTGGCCGAGCAAGCGCTTGGTTGATAGTC	Oligonucleotides used in EMSAs
	Reverse	GACTATCAACCAAGCGCTTGCTCGGCCAGC	Oligonucleotides used in EMSAs
*Rv3560c/Rv3561*	Forward	GCTAACCTACCAAGCACTTGCTTTGTTAGG	Oligonucleotides used in EMSAs
	Reverse	CCTAACAAAGCAAGTGCTTGGTAGGTTAGC	Oligonucleotides used in EMSAs

*The locations of the motifs are underlined; bold type indicates the start (ATG) and stop (TCA) codons of the *kstR2* gene.

**Table 3.** Instances of the KstR2 motif in *M. smegmatis*, *M. tuberculosis* and *R. jostii* nd, Not done; +, positive EMSA result. Underlining represents the motif location.

**Motif sequence**	***P*-value**	**Flanking genes**	**EMSA**
***M. smegmatis***			
TACCAAGCAAGTGCTTGCTTAGGT	2.0e−08	*MSMEG_6000/MSMEG_6001*	nd
TACCAACCAAGCACTTGCTAGGTC	5.6e−14	*MSMEG_6009/MSMEG_6010*	nd
AACCTACCAAGCACTTGCTTTGTT	3.6e−08	*MSMEG_6012/MSMEG_6013*	nd
***M. tuberculosis***			
TACCAAGCAAGTGCTTGCTTAGGT	2.0e−08	*Rv3549c/Rv3550*	+
TATCAACCAAGCGCTTGCTCGGCC	2.0e−15	*Rv3557c/Rv3558*	+
AACCTACCAAGCACTTGCTTTGTT	3.6e−08	*Rv3560c/Rv3561*	+
***R. jostii***			
ACCGAACCAAGCGATTGCTTGGTA	1.9e−07	*ro04652/04653*	nd
GCCCTAACAAGCGCTTGGTTGATA	7.0e−14	*ro04598*	nd
TACCTAGCAAGTGCTTGGTAGGTT	3.9e−07	*ro04595/04596*	nd

**Table 4.** Expression analysis of Δ*kstR2*_Msm_ and the *kstR2* regulon Genes in bold type are part of the *kstR2* regulon.

**Direction of transcription**	***M. smegmatis* gene**	**Fold change**	***P*-value**	***M. tuberculosis* gene**	**Function**
	*MSMEG_1061* genes	−40.4	5.0e−03	–	Phosphohydrolase
	*MSMEG_1112*	−45.2	2.0e−02	–	Putative aconitase hydratase
**↑**	MSMEG_5999*	**140.3**	**1.0e−04**	***Rv3548c***	**Probable short-chain-type dehydrogenase/reductase**
**↑**	MSMEG_6000*	**2017.0**	**1.2e−07**	***Rv3549c***	**Probable short-chain-type dehydrogenase/reductase**
**↓**	MSMEG_6001*	**250.7**	**2.0e−05**	***Rv3550***	**Probable enoyl-CoA hydratase**
**↓**	MSMEG_6002*	**150.5**	**7.0e−05**	***Rv3551*†‡§**	**Possible CoA-transferase (alpha subunit)**
**↓**	MSMEG_6003*	**110.6**	**8.2e−05**	***Rv3552*†§**	**Possible CoA-transferase (beta subunit)**
**↓**	MSMEG_6004*	**5.2**	**1.9e−01**	***Rv3553***	**Possible oxidoreductase**
↓	*MSMEG_6005*	2.3	5.5e−01	–	Hypothetical protein
↓	*MSMEG_6006*	1.1	9.6e−01	–	Hypothetical protein
↑	*MSMEG_6007*	−1.1	9.6e−01	–	Cation diffusion transporter||
**↑**	MSMEG_6008*	**88.8**	**8.2e−05**	***Rv3556c*†‡§**	**Probable acetyl-CoA acetyltransferase**
**↑**	MSMEG_6009*	**−1.2**	**9.0e−01**	***Rv3557c*§**	**Transcriptional regulatory protein (kstR2)**
↓	*MSMEG_6010*	−3.0	3.0e−01	–	Hypothetical protein
**↑**	MSMEG_6011*	**3.2**	**3.4e−01**	***Rv3559c***	**Possible oxidoreductase**
**↑**	MSMEG_6012*	**173.6**	**2.0e−05**	***Rv3560c*†‡**	**Probable acyl-CoA dehydrogenase**
**↓**	MSMEG_6013*	**2.2**	**5.1e−01**	***Rv3561*§**	**Probable fatty-acid-CoA ligase**||
**↓**	MSMEG_6014*	**1.6**	**7.6e−01**	***Rv3562*§**	**Probable acyl-CoA dehydrogenase**
**↓**	MSMEG_6015*	**15.5**	**2.6e−02**	***Rv3563*†‡**	**Probable acyl-CoA dehydrogenase**
**↓**	MSMEG_6016*	**9.5**	**9.4e−02**	***Rv3564***	**Probable acyl-CoA dehydrogenase**
**↓**	MSMEG_6017*	**100.8**	**9.5e−01**	***Rv3565***	**Possible aspartate aminotransferase**
	*MSMEG_6197*	−15.9	4.0e−02	–	Diaminopimelate decarboxylase

*Orthologous genes de-repressed by cholesterol in *Rhodococcus jostii* RHA1. †Essential in macrophages according to transposon site hybridization (TraSH) studies (Rengarajan *et al.*, 2005). ‡Essential in mice according to TraSH studies (Sassetti & Rubin, 2003). §Induced in macrophages (Schnappinger *et al.*, 2003). ||*M. smegmatis* gene contains a frameshift mutation – pseudogene?

## Abbreviations

- EMSA, electrophoretic mobility shift assays
- OADC, oleic acid–albumin–dextrose–catalase
- RTq-PCR, quantitative RT-PCR
